# Supplementary material for: Converging and evolving immuno-genomic routes toward immune escape in breast cancer
Source: Nat Commun. 2024 Feb 21;15:1302. doi: 10.1038/s41467-024-45292-1 (PMC10882008; doi:10.1038/s41467-024-45292-1)
Supplement: Supplementary file 1 — Supplementary Information [file 41467_2024_45292_MOESM1_ESM.pdf]

Supplementary Figure 1

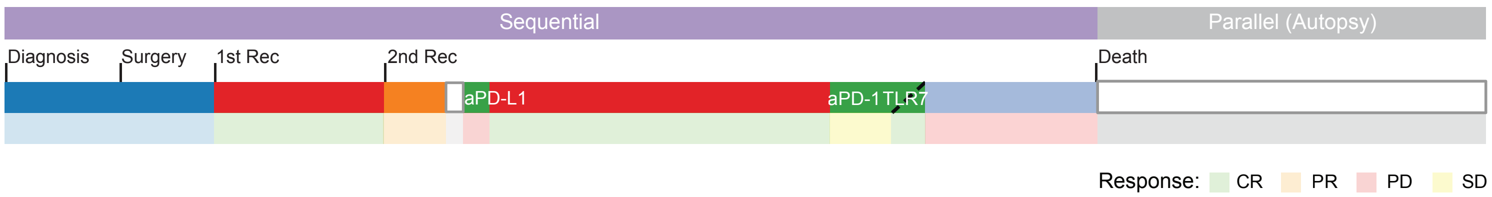

Index patient systemic therapies

Therapy	Days from Diagnosis	Best Response
Anthracline-taxane (Neoadjuvant)	~191	pCR
Cisplatin-based (Recurrence chest wall)	373	CR
Bevacizumab-based (Recurrence chest wall)	~666	PR
Checkpoint inhibitor (1st line)	799	PD
Cisplatin-based (2nd line)	~854	CR
Checkpoint inhibitor + cisplatin-based	~1519	SD
Checkpoint inhibitor + TLR7 agonist	~1519	CR*
Other	~1687	PD

Platform Analysis

Blood: Single-cell RNA-seq
IFNγ ELISpot

Plasma: ctDNA high depth targeted sequencing
Multiplexed Proteomics

Tumor: Sequential and parallel (Bulk)
★ TCRβ-seq
M CD8, CD4, CD3, Ki67 IHC & TILs
○ WES / high depth targeted sequencing
○ Bulk RNA-seq / 770 immuno-oncology gene expression, Multiplexed Proteomics

Data Analysis

TCRα/β and single-cell immunophenotyping

TCRβ networks

Gene expression-based immuno analysis

HLA Class I and Class II gene expression

Allele-specific HLA Class I loss

Mutation / Neoantigen load, clonality and time

Supplementary Figure 1. Complementary schematics of the index TNBC patient. Systemic therapies administered and the analyses performed for the index TNBC patient.
aPD-L1, anti-programmed death-ligand 1 monoclonal antibody; aPD-1, anti-programmed cell death protein 1 monoclonal antibody; CR, complete response; ctDNA, circulating tumor DNA; ELISpot, Enzyme-Linked ImmunoSpot; IHC, immunohistochemistry; M, metastasis; pCR, pathologic complete response; RNA-seq, RNA sequencing; PR, partial response; PD, progressive disease; Rec, recurrence; SD, stable disease; TCR, T cell receptor sequencing; TCRβ, TCR beta chain; TILs, tumor-infiltrating lymphocytes; TLR7, Toll-like receptor 7; WES, whole-exome sequencing. (*) Transient complete response to pembrolizumab plus TLR7 agonist (days ~1637-1687).

Supplementary Figure 2

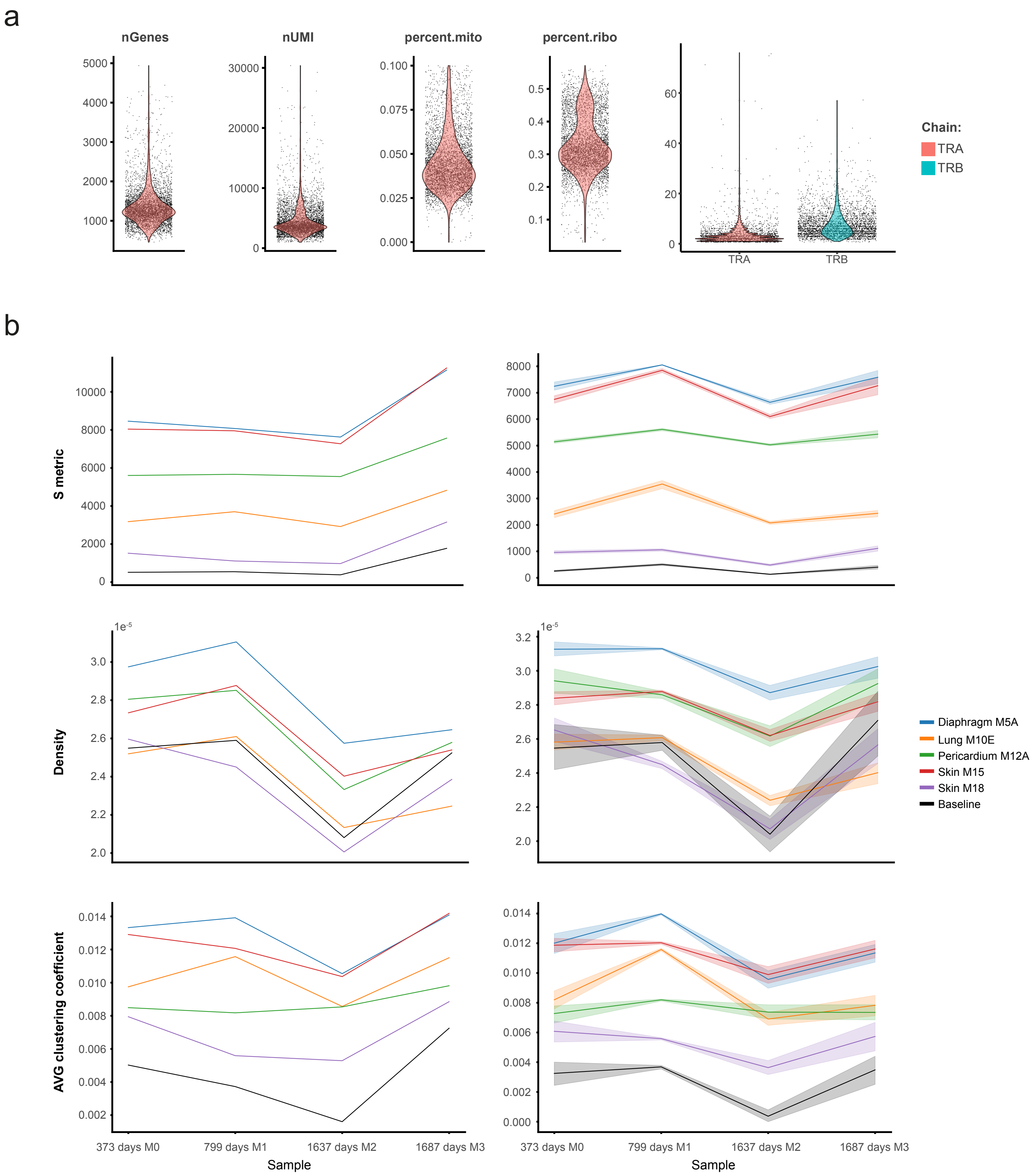

c

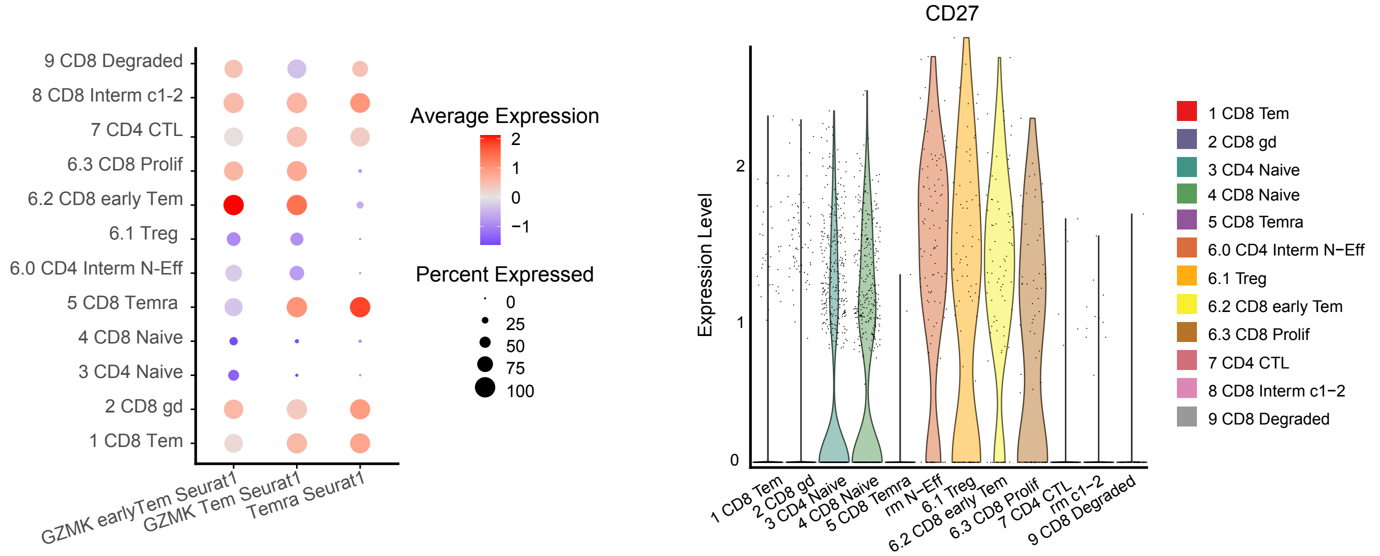
9 CD8 Degraded
8 CD8 Interm c1-2
7 CD4 CTL
6.3 CD8 Prolif
6.2 CD8 early Tem
6.1 Treg
6.0 CD4 Interm N-Eff
5 CD8 Temra
4 CD8 Naive
3 CD4 Naive
2 CD8 gd
1 CD8 Tem
GZMK earlyTem Seurat1
GZMK Tem Seurat1
Temra Seurat1
Average Expression
2
1
0
−1
Percent Expressed
0
25
50
75
100
CD27
Expression Level
2
1
0
1 CD8 Tem
2 CD8 gd
3 CD4 Naive
4 CD8 Naive
5 CD8 Temra
rm N-Eff
6.1 Treg
6.2 CD8 early Tem
6.3 CD8 Prolif
7 CD4 CTL
rm c1−2
9 CD8 Degraded
1 CD8 Tem
2 CD8 gd
3 CD4 Naive
4 CD8 Naive
5 CD8 Temra
6.0 CD4 Interm N−Eff
6.1 Treg
6.2 CD8 early Tem
6.3 CD8 Prolif
7 CD4 CTL
8 CD8 Interm c1−2
9 CD8 Degraded

d

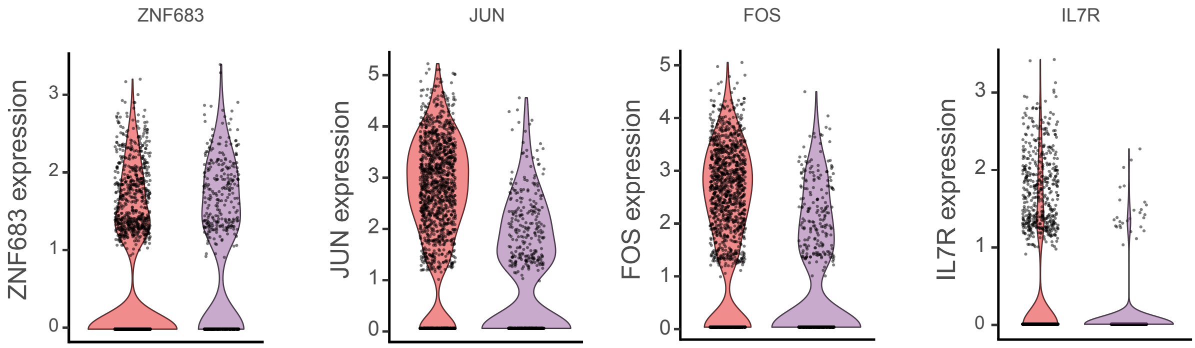
ZNF683
JUN
FOS
IL7R
ZNF683 expression
JUN expression
FOS expression
IL7R expression
0
1
2
3
4
5

e

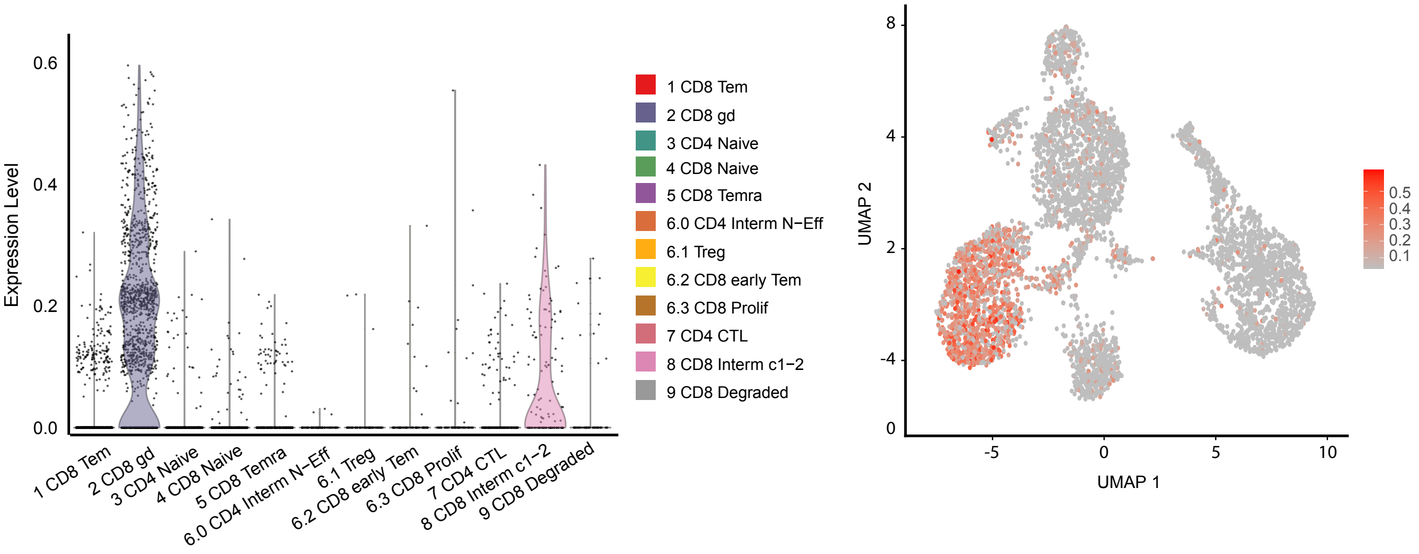
Expression Level
0.6
0.4
0.2
0.0
1 CD8 Tem
2 CD8 gd
3 CD4 Naive
4 CD8 Naive
5 CD8 Temra
6.0 CD4 Interm N-Eff
6.1 Treg
6.2 CD8 early Tem
6.3 CD8 Prolif
7 CD4 CTL
8 CD8 Interm c1−2
9 CD8 Degraded
1 CD8 Tem
2 CD8 gd
3 CD4 Naive
4 CD8 Naive
5 CD8 Temra
6.0 CD4 Interm N−Eff
6.1 Treg
6.2 CD8 early Tem
6.3 CD8 Prolif
7 CD4 CTL
8 CD8 Interm c1−2
9 CD8 Degraded
UMAP 2
8
4
2
-4
0
-5
0
5
10
UMAP 1
0.5
0.4
0.3
0.2
0.1

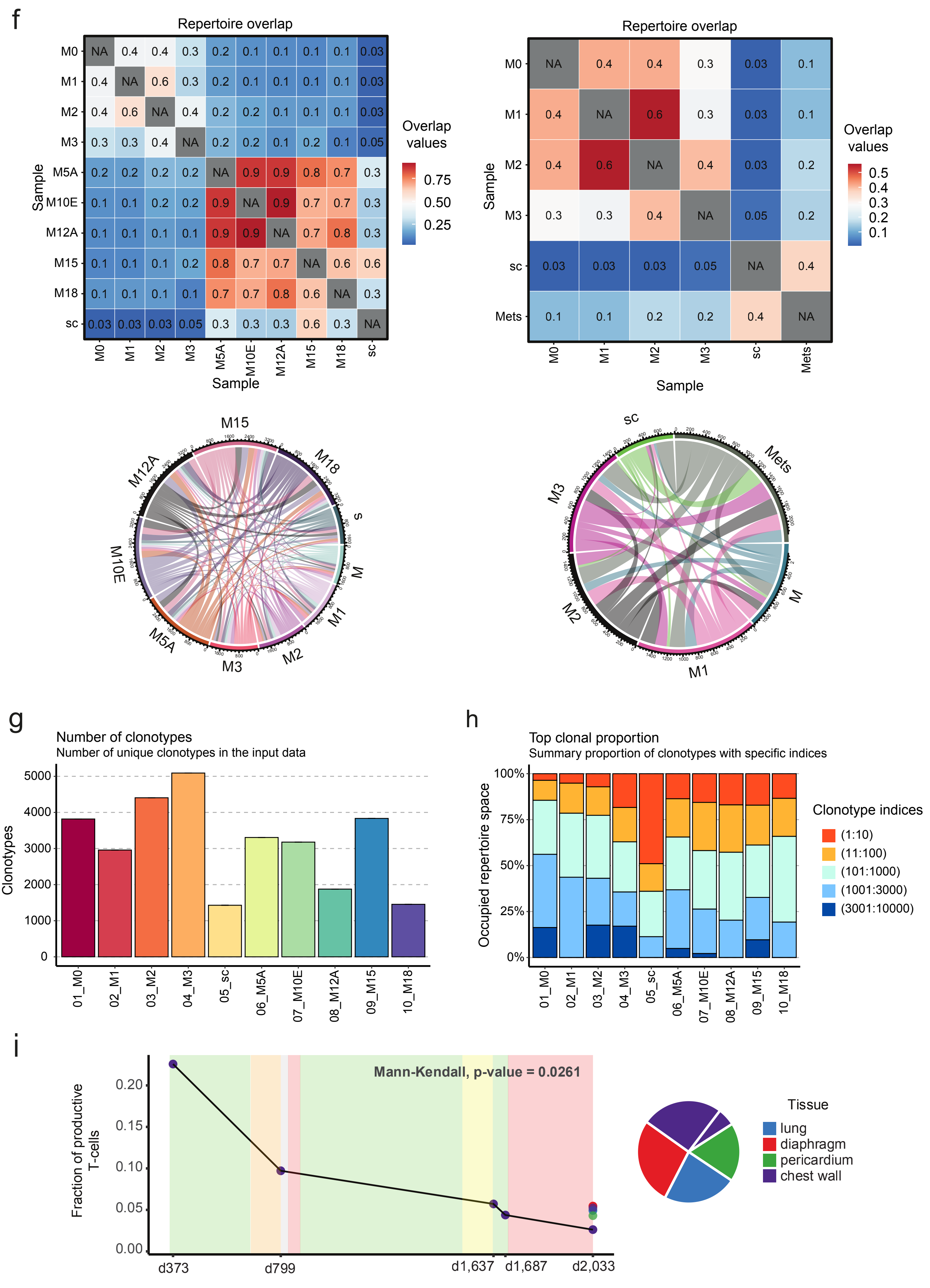

f
Repertoire overlap
Sample
Overlap values
0.75
0.50
0.25
M0
M1
M2
M3
M5A
M10E
M12A
M15
M18
sc
Repertoire overlap
Sample
Overlap values
0.5
0.4
0.3
0.2
0.1
Mets
g
Number of clonotypes
Number of unique clonotypes in the input data
Clonotypes
01_M0
02_M1
03_M2
04_M3
05_sc
06_M5A
07_M10E
08_M12A
09_M15
10_M18
h
Top clonal proportion
Summary proportion of clonotypes with specific indices
Occupied repertoire space
Clonotype indices
(1:10)
(11:100)
(101:1000)
(1001:3000)
(3001:10000)
i
Mann-Kendall, p-value = 0.0261
Fraction of productive T-cells
d373
d799
d1,637
d1,687
d2,033
Tissue
lung
diaphragm
pericardium
chest wall

Supplementary Figure 2. Single-cell RNA and T cell receptor sequencing of T cells from the peripheral blood and metastases of the index patient. Source data for all panels are provided as a Source Datafile.

a, Quality control figures (nGenes, nUMI, mitochondrial percentage, ribosomal genes, TCRa/TCRb chain read counts and sequencing depth) are shown. **b,** Plots depicting density, average clustering coefficient and S-metric (left side). Average clustering coefficient and S-metric's values increased in later time points sequential metastases and when they were computationally integrated with postmortem parallel multiregion metastases. As a means of validation, bootstrap analysis was performed by downsampling and reproducing the networks and recomputing the metrics (right). **c,** Mean expression for a set of signatures from ref[1] representing the T cell states of differentiation (left). Violin plot showing CD27 overexpression across the T cell population clusters (right). **d,** Distribution of ZNF683**,** AP-1 transcription factors JUN and FOS and IL7R memory marker among cluster 1 (CD8Tem) and cluster 5 (CD8 Temra). Violin plot showing enrichment score for each cell on each cluster for γδ markers obtained from Pizzolato et al, 2019**. e,** UMAP visualization of all sequenced cells colored by the mentioned γδ enrichment score. **f,** TCRβ CDR3 repertoire overlap and circus plots among on-treatment, multiregion metastases (average is shown) and single T cell from peripheral blood. In the circus plots, the axis on each sample is the total number of clones shared between all the samples. The ribbons connect the two samples from which we have observed shared clones and the size of the ribbons are the number of clones shared. **g,** Clonotype counts of each metastatic lesion and peripheral blood are depicted. **h,** Top clonal proportion. **i,** Longitudinal changes in the fraction of productive T cells on therapy and among autopsy tissues represented in the pie chart (N=9 tumors). Mann-Kendall test showing that decreasing trend over time is statistically significant.

Supplementary Figure 3

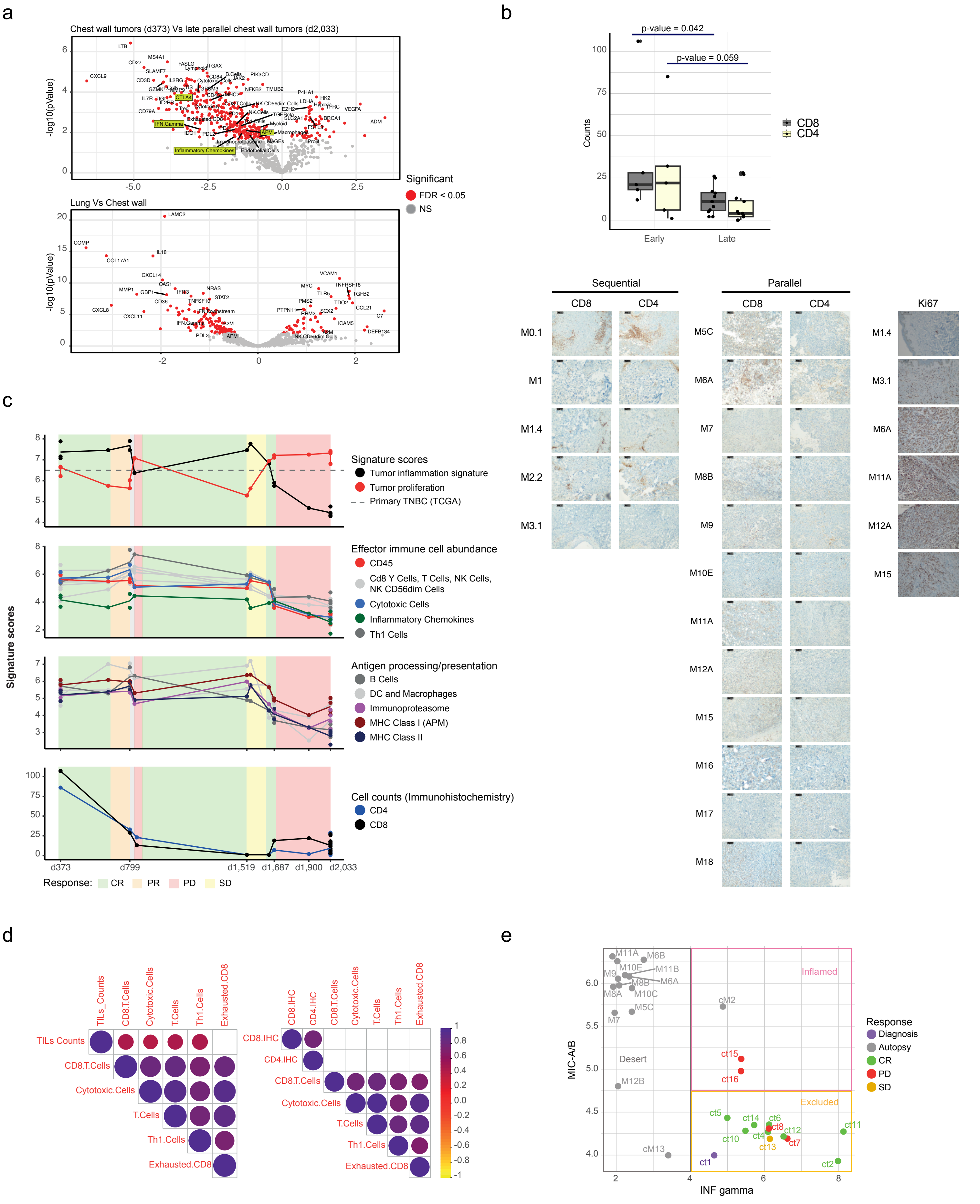

f

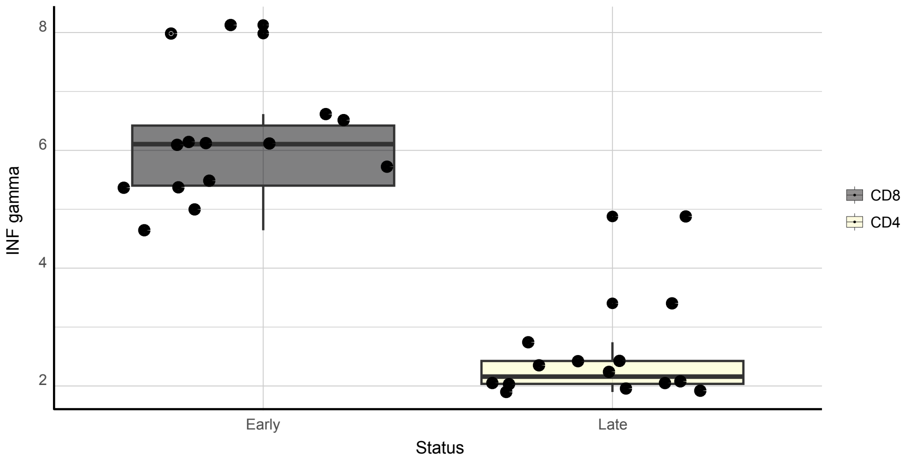

g

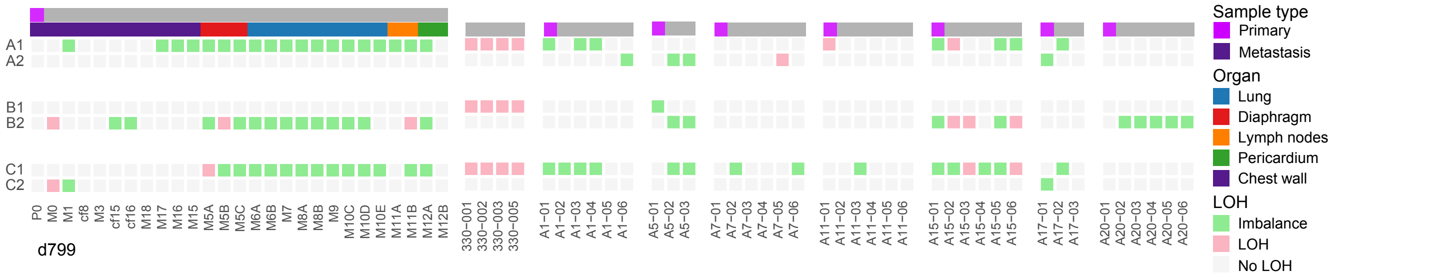

Supplementary Figure 3. Key players revealing immune escape in the immune microenvironment. Source data for all panels are provided as a Source Data file.

a, Multiplexed gene expression profiles identified 283 statistically significant downregulated genes among immunotherapy naïve chest wall tumors and postmortem parallel tumors (n=7 tumors). IFNy signaling pathway, antigen processing machinery (APM) signaling, immune cell abundance, immune checkpoints, inflammatory chemokines are depicted. FDR < 0.05 is considered statistically significant. Multiplexed gene expression profi es among chest wall tumors and lung tumors sampled at autopsy. FDR < 0.05 was considered statistically significant. **b**, Immunohistochemical analyses of CD4 and CD8 T cells and TILs analyses in selected biopsies across on sequential and parallel multiregion metastases (t statistics from a generalized linear model, p-values < 0.05 are statistically significant). N=18 biological independent samples. Representative micrographs were taken with a 100X objective; scale bars, 0.2 µm. Ki67 expression by IHC in selected biopsies across on treatment phase and parallel multiregion metastases. Scale bars, 200 µm. **c**, Longitudinal monitoring of tumor inflammation signature, tumor proliferation scores, effector immune cell abundance, antigen processing and presentation gene expression scores. Gray dotted lines represent the median of tumor inflammation signature (TIS) scores derived from the primary TNBCs of the TCGA. **d**, Spearman correlation matrix highlighting the concordance between pathology TILs counts (left panel) or CD4+ and CD8+ T cell counts (IHC) and mRNA-based T cell signature estimates. Purple color represents positive correlation whereas yellow represents negative correlations. Color intensity and size of the circle are proportional to the correlation coefficients which are depicted in the legend to the right. Blank squares correspond to non-significant (p-values > 0.05) correlations. N=18 biological independent samples. **e**, Validation of the gene expression-based immunophenotype status with Multiplexed Proteomic Assay (IFNy and MIC A/B (i.e., HLA-A and B)) across 19 parallel multiregional metastases that mapped mostly to the desert phenotype (IFNylow, MIC A/B high/low). **f**, IFNy levels in early versus late metastases as per Multiplexed Proteomics Assay. N=35 biological independent samples. Wilcoxon rank sum exact test was used. Boxplot limits indicate the interquartile range (IQR; 25th to 75th percentile), with a center line indicating the median. Whiskers show the value ranges up to 1.5 x IQR above the 75th or below the 25th percentile with outliers beyond those ranges shown as individual points. **g**, HLA class I loss and imbalance for the index patient (M1, and 20 parallel metastases) and the cohort of 10 TNBC patients showing primary and multiregion metastases. HLA loss and imbalance were assigned as described in Methods. Case 330 does not have the primary breast cancer depicted.

Supplementary Figure 4

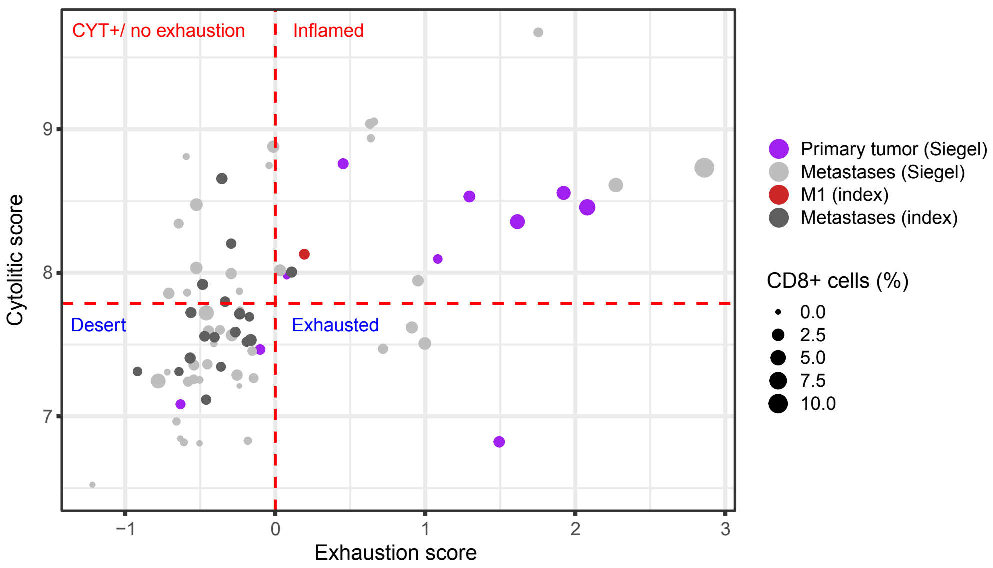

Supplementary Figure 4. T cell exhaustion score and soluble PD-L1 and IFNy follow the immune evolution during metastatic progression. Source data are provided as a Source Data file.

T cell exhaustion metagene score, cytolytic score and CD8+ T cell signatures across the samples of the index case (M1 and multiregion metastases) and the TNBC cohort (primary samples and multiregion metastases). Note that the majority of parallel multiregion metastases are mapped under "desert tumors" (T cell exhaustion metagene score ^{Low}/cytolytic score ^{Low} / CD8+ ^{Low}). CYT, cytolytic.

Supplementary Figure 5

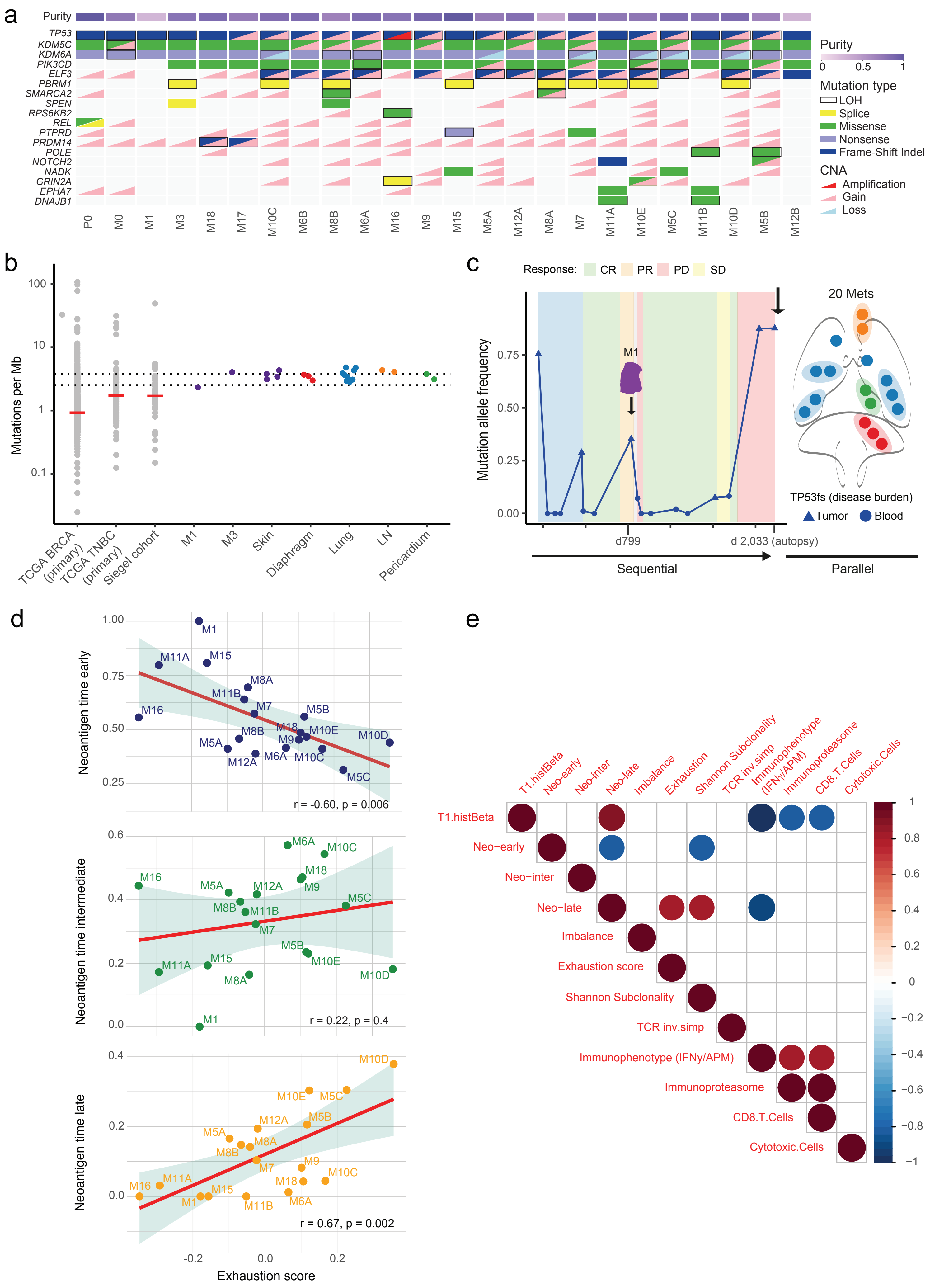

f

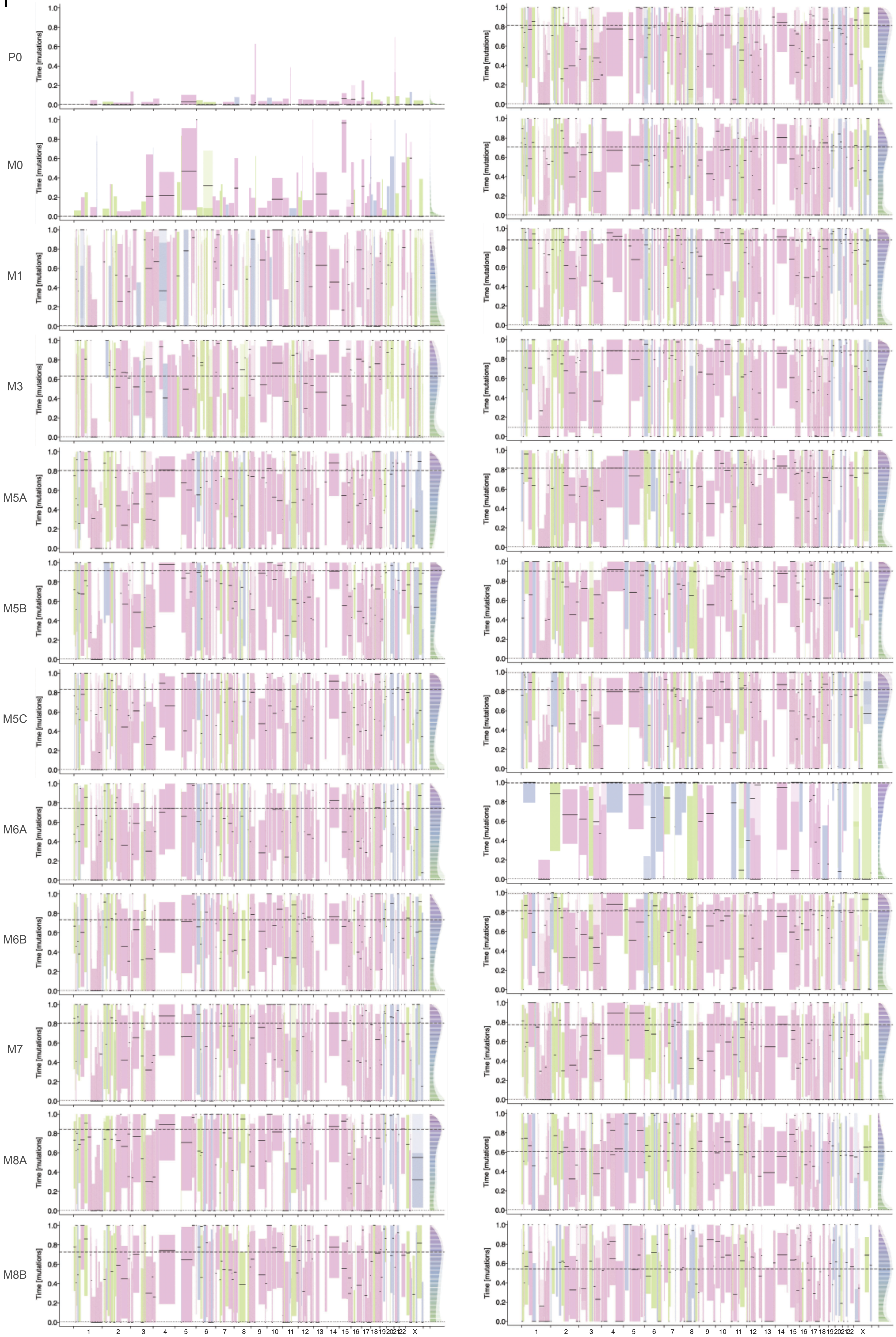
P0
M0
M1
M3
M5A
M5B
M5C
M6A
M6B
M7
M8A
M8B
M9
M10C
M10D
M10E
M11A
M11B
M12A
M12B
M15
M16
M17
M18
Time [mutations]
1 2 3 4 5 6 7 8 9 10 11 12 13 14 15 16 17 18 19 20 21 22 X

g

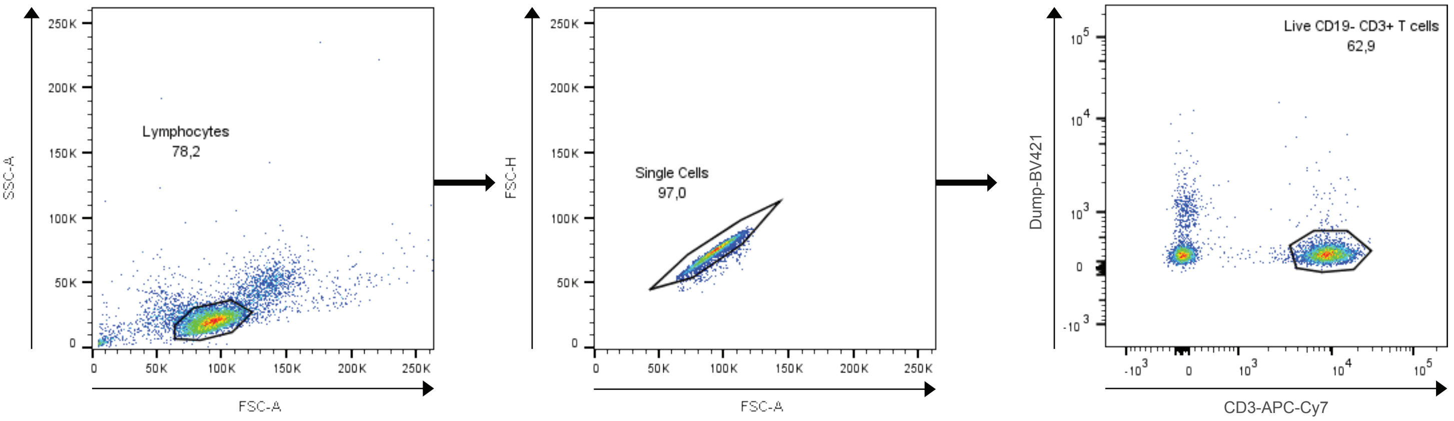

Supplementary Figure 5. Genomic landscape and Neonatigen timing of sequential and parallel metastases of the index patient. Source data for all panels are provided as a Source Data file.

a, Tile plots showing the repertoire of mutations and copy number alterations for the index patient. CNA, copy number alteration. **b**, TMB from the TCGA TNBC cohort2,3 (105 primary tumor samples classified as per ER, PR, HER2 negative) is higher than the TCGA BRCA cohort (1,009 primary breast tumor samples). M0, M1, M3 chest wall tumors and postmortem parallel metastases are mapped along the inferred TMB of the TCGA cohorts. Using WES, an average of 855 (range 559-1,095) nonsynonymous somatic mutations were identified in metastases (i.e., M1, M3, multiregion metastases) with a mean coverage of > 100x, including 733 single nucleotide variants (SNVs) (range 396-926) and 130 indels (range 82-165). The color in metastases refers to the organ of origin. **c**, Personalized digital PCR targeting TP53 T256fs mutation across eleven serial blood samples and six tissue biopsies (diagnostic primary breast tumor and five chest wall lesions covering non-metastatic and metastatic phases of disease). **d**, Correlation between Exhaustion score and Neoantigen Time. N=18 biologically independent samples. Spearman correlation used, P-value < 0.05 is considered statistically significant. **e**, Spearman correlation matrix of key immune and genomic parameters and Neoantigen Time among sequential and parallel multiregion metastases of the index case. N=24 biologically independent samples. Red color represents positive correlation whereas blue represents negative correlations. Color intensity and size of the circle are proportional to the correlation coefficients, which are depicted in the legend to the right. Blank squares correspond to non-significant (p-values > 0.05) correlations. **f**, Plots show the estimated mutation time of primary and secondary copy number gains (shaded). Boxes denote 95% CIs. Blue, mono-allelic gains (N:1), pink, CN-LOH/gain plus loss (N:0) and, green, bi-allelic gains(N:2). **g**, Fluorescence activated cell sorting (FACS) sequential gating/sorting strategies.

Supplementary Table 1

Disease status	Therapy	Best response	Days from diagnosis
Neoadjuvant	Anthracycline plus taxane	Pathological complete response	~191
	Mastectomy		
Recurrence #1 (loco-regional- right chest wall and axillary glands)	Cisplatin plus gemcitabine	**Complete response**	~373-666
	Surgery		
	Loco-regional radiotherapy		
Recurrence #2 (right chest wall)	Docetaxel plus bevacizumab	Stable disease	~666
	Capecitabine plus bevacizumab	Partial response	
Metastatic disease - First line	Atezolizumab (anti-PDL1)	Progressive disease	799
Metastatic disease - Second line	Cisplatin plus gemcitabine ->maintenance with cyclophosphamide	**Complete response**	~854-1519
Recurrence #3 (right chest wall)	Pembrolizumab plus cisplatin plus gemcitabine plus methotrexate --> weekly cisplatin	Stable disease	~1519-1637
New systemic therapy line	Pembrolizumab plus Toll-like receptor (TLR) 7 agonist (topical)	**Transient complete response**	~1637-1687
New systemic therapy line	Palbociclib folowed by cyclophosphamide	Progressive disease	~1687-2033
New systemic therapy line	Pegylated liposomal doxorubicin	Progressive disease	
New systemic therapy line	Cisplatin plus gemcitabine	Progressive disease	
New systemic therapy line	Paclitaxel plus bevacizumab	Progressive disease	
New systemic therapy line	Eribulin	Stable disease	
Patient passed away			2033

Supplementary Table 1: TNBC patient treatments over 2,033 days.

Supplementary Table 2

Blood_count_date	total_leukocytes	abs_neutrophils	abs_lymphocytes	neutrophils-to-lymphocytes ratio
25/06/15	2600	1400	900	1.6
15/07/15	2400	1300	700	1.9
10/08/15	2600	1500	700	2.1
23/09/15	3300	2400	700	3.4
03/11/15	2800	2000	500	4.0
10/12/15	3800	2800	600	4.7
08/01/16	2400	1700	500	3.4
16/02/16	2100	1400	400	3.5
29/03/16	2600	1800	500	3.6
12/05/16	2300	1400	600	2.3
11/06/16	2400	1500	500	3.0
02/08/16	2300	1600	400	4.0
05/09/16	2000	1300	400	3.3
13/12/16	2100	1300	500	2.6
24/01/17	2600	2000	300	6.7
03/02/17	2000	1200	600	2.0
21/03/17	1700	1000	400	2.5
25/04/17	1200	600	400	1.5
11/05/17	1600	800	500	1.6
26/06/17	1400	600	500	1.2
01/08/17	500	100	200	0.5
31/08/17	2400	1100	700	1.6
28/09/17	11300	9000	800	11.3
27/10/17	2500	900	500	1.8
24/11/17	9600	8200	1200	6.8
28/12/17	4400	3000	800	3.8
31/01/18	4500	3200	700	4.6
19/02/18	3700	2800	700	4.0
27/02/18	5400	3700	900	4.1
12/03/18	8800	8200	300	27.3
09/04/18	4400	3500	800	4.4
30/04/18	4200	2600	1200	2.2
09/05/18	2600	1700	500	3.4
31/05/18	3800	2500	800	3.1
08/06/18	5100	3200	800	4.0
12/06/18	5800	5100	200	25.5

Supplementary Table 2: Clinical peripheral blood counts of the index patient.

Supplementary Reference

1. Zheng, L. *et al.* Pan-cancer single-cell landscape of tumor-infiltrating T cells. *Science (80-.).* **374**, (2021).
2. Yates, L. R. *et al.* Subclonal diversification of primary breast cancer revealed by multiregion sequencing. *Nat. Med.* **21**, 751–759 (2015).
3. Angelova, M. *et al.* Evolution of Metastases in Space and Time under Immune Selection. *Cell* **175**, 751-765.e16 (2018).
