# Supplementary material for: Converging and evolving immuno-genomic routes toward immune escape in breast cancer
Source: Nat Commun. 2024 Feb 21;15:1302. doi: 10.1038/s41467-024-45292-1 (PMC10882008; doi:10.1038/s41467-024-45292-1)
Supplement: Supplementary file 3 — Description of Additional Supplementary Files [file 41467_2024_45292_MOESM3_ESM.pdf]

Description of Additional Supplementary Files

File Name: Supplementary Data 1
Description: Metadata, sample and experimental characteristics.
