# Supplementary material for: Converging and evolving immuno-genomic routes toward immune escape in breast cancer
Source: Nat Commun. 2024 Feb 21;15:1302. doi: 10.1038/s41467-024-45292-1 (PMC10882008; doi:10.1038/s41467-024-45292-1)
Supplement: Supplementary file 5 — Reporting Summary [file 41467_2024_45292_MOESM5_ESM.pdf]

nature research

Corresponding author(s): L. De Mattos-Arruda NCOMMS-23-38969A

Last updated by author(s): Jan 9, 2024

Reporting Summary

Nature Research wishes to improve the reproducibility of the work that we publish. This form provides structure for consistency and transparency in reporting. For further information on Nature Research policies, see our Editorial Policies and the Editorial Policy Checklist.

Statistics

For all statistical analyses, confirm that the following items are present in the figure legend, table legend, main text, or Methods section.

n/a	Confirmed	
☐	☒	The exact sample size (*n*) for each experimental group/condition, given as a discrete number and unit of measurement
☐	☒	A statement on whether measurements were taken from distinct samples or whether the same sample was measured repeatedly
☐	☒	The statistical test(s) used AND whether they are one- or two-sided<br>*Only common tests should be described solely by name; describe more complex techniques in the Methods section.*
☐	☒	A description of all covariates tested
☐	☒	A description of any assumptions or corrections, such as tests of normality and adjustment for multiple comparisons
☐	☒	A full description of the statistical parameters including central tendency (e.g. means) or other basic estimates (e.g. regression coefficient) AND variation (e.g. standard deviation) or associated estimates of uncertainty (e.g. confidence intervals)
☐	☒	For null hypothesis testing, the test statistic (e.g. *F*, *t*, *r*) with confidence intervals, effect sizes, degrees of freedom and *P* value noted<br>*Give P values as exact values whenever suitable.*
☐	☒	For Bayesian analysis, information on the choice of priors and Markov chain Monte Carlo settings
☒	☐	For hierarchical and complex designs, identification of the appropriate level for tests and full reporting of outcomes
☐	☒	Estimates of effect sizes (e.g. Cohen's *d*, Pearson's *r*), indicating how they were calculated

Our web collection on statistics for biologists contains articles on many of the points above.

Software and code

Policy information about availability of computer code

Data collection	No software was used for data collection
Data analysis	Single-cell mapping: CellRanger v.3.1.0<br>Single-cell analyses: Seurat v3.2.1<br>TCR inference from bulk RNA-seq: MiXCR v3.0.12<br>Demultiplexing: Illumina's bcl2fastq2 v.2.17 software<br>Filtering fastp v.0.19.5<br>Alignment: BWA v0.7.7 to the reference hg38<br>Mutation calling: MuTect2 v4.1.0.0<br>Mean coverage calculation: CollectWgsMetrics by Picard v4.0<br>Mutation annotation: VEP release 87<br>Visualization: IGV v2.4<br>RNA-seq trimming: Trimmomatic v0.27<br>RNA-seq gene expression: Kallisto v0.46.0<br>Cancer Cell Fraction: PyClone-vi<br>Segmentation: Sequenza v3.0.0<br>HLA typing HLA-HD v1.4.0<br>Neoantigen prediction: NeoPredPipe 1.1<br>Molecular timing: MutationTimeR v1.00.2<br>Clonal evolution: ClonEvol v0.99.11<br>HLA Loss: LOHHLA<br>Statistical analysis: R 3.6.3<br>Libraries "ggplot2", "ggpubr", "dplyr", "ggthemes", "pheatmap", "ggupset", "reshape2", "lemon", "tidyverse", "corrplot", "cluster", "Biobase",

"qvalue", “seurat”, “fishplot”, “mutationtimer”, "cluster", "DESeq2", "RColorBrewer".
Circa plots: pyCircos
Python libraries: numpy (1.19.4) , pandas (1.1.4), scanpy (1.6.1), matplotlib (3.2.2), Biopython (1.76)

For manuscripts utilizing custom algorithms or software that are central to the research but not yet described in published literature, software must be made available to editors and reviewers. We strongly encourage code deposition in a community repository (e.g. GitHub). See the Nature Research guidelines for submitting code & software for further information.

Data

Policy information about availability of data

All manuscripts must include a data availability statement. This statement should provide the following information, where applicable:

- Accession codes, unique identifiers, or web links for publicly available datasets
- A list of figures that have associated raw data
- A description of any restrictions on data availability

Data Availability. The data generated in this study (whole exome sequencing and RNAseq of tumor specimens, plus single cell RNAseq and single cell TCRseq of peripheral blood T cells) have been deposited in the European Genome-Phenome Archive (EGA), under restricted access to protect patient information, under the accession code EGAS00001004956. Access will be granted by application to the Data Access Committee (DAC, EGAC50000000074) with responses addressed within 14 working days. Access will be granted for appropriate use and will be governed by the provisions laid out in the associated informed consent for each cohort or collection, and the terms contained in the Data Access Agreement.

The Siegel data10 are available under restricted access for the project ID #19480 (Tumor microenvironment analysis on metastatic breast cancer) under Tiezzi DG responsibility as the Principal investigator, access was obtained by NIH DAC approval at the NCBI dbGaP system. Sample information for RNA-seq and DNA-seq fastQ runs, including the clinical information, was downloaded from the NCBI’s dbGAP (phs000676.v1.p1)10. Further DNA and RNA-seq data used in this study are available in the EGA database under accession code EGAS0000100270311. The remaining data are available within the Article, Supplementary Information or Source Data file. Source data are provided with this paper.

Code Availability. The computational scripts to process the data and plot figures are available at Blanco-Heredia, J. No Title. jblancoheredia/genomic_immune_tnbc_2024: 11DEC23 (v0.0). Zenodo. https://doi.org/10.5281/zenodo.10359740 (2023).

Field-specific reporting

Please select the one below that is the best fit for your research. If you are not sure, read the appropriate sections before making your selection.

☒ Life sciences ☐ Behavioural & social sciences ☐ Ecological, evolutionary & environmental sciences

For a reference copy of the document with all sections, see nature.com/documents/nr-reporting-summary-flat.pdf

Life sciences study design

All studies must disclose on these points even when the disclosure is negative.

Sample size

A total of 104 specimens for the 12 patients were analyzed: 11 primary tumors, 16 on-treatment metastases, 11 serial blood samples and 66 postmortem metastases.

INDEX PATIENT: We evaluated the matched normal, primary tumor (N=1), sequential (longitudinal) biopsies (n=16) and blood samples (n=11) and parallel multiregion tumor (n=20) during 2,033-day follow-up of the index TNBC patient (n=48 in total).

VALIDATION COHORT: 11 TNBC patients included in the study as validation cohort and subjected to re-analysis of their WES and bulk-RNA seq data for primary tumors (n=10) and parallel multiregion metastases (n=46).
Siegel, M. B. et al. Integrated RNA and DNA sequencing reveals early drivers of metastatic breast cancer. J. Clin. Invest. 128, (2018).
De Mattos-Arruda, L. et al. The Genomic and Immune Landscapes of Lethal Metastatic Breast Cancer. Cell Rep. (2019)

The 1,058 primary breast cancer (BRCA) cases from The Cancer Genome Atlas (TCGA), including 113 TNBC cases were evaluated for their inflamed signatures and tumor mutation burden.

Data exclusions	Single T cell RNA-seq and TCR data analysis: A quality control on the cells was applied prior to downstream analysis. Cells with <200 expressed genes, <1000 unique molecular identifier (UMI) counts or >10% of expressed mitochondrial genes were excluded (likely degraded or broken cells). In addition, cells with >5000 expressed genes or >40000 UMI counts were removed from the analysis to exclude potential doublets.<br><br>Whole Exome sequencing data: MuTect2 (v4.1.0.0) was used two extra filters in the functionFilterMutectCalls were added a)min-allele-fraction settled to 0.05 and b) unique-alt-read-count to keep mutations supported by 10 or more reads. Output outside this range was excluded in further analyses.<br><br>Nanostring gene expression profiling: Samples in which the mean expression of housekeeper genes fell below 5 (32 raw counts) were discarded.<br><br>RNA-seq: RNA samples with RIN (RNA integrity) < 4 and rRNA (ribossomal RNA) ratio < 1 were discarded. FASTQ files of RNA-seq reads (paired-end) from tumor samples were pre-processed with Trimmomatic (v0.27) to remove Illumina adapter sequences, trim low-quality read ends, crop long-reads to a maximum length, and discard short reads.<br><br>ddPCR: Only samples with >10,000 droplets were accepted as valid.
Replication	n/a
Randomization	n/a
Blinding	n/a

Reporting for specific materials, systems and methods

We require information from authors about some types of materials, experimental systems and methods used in many studies. Here, indicate whether each material, system or method listed is relevant to your study. If you are not sure if a list item applies to your research, read the appropriate section before selecting a response.

Materials & experimental systems

n/a	Involved in the study	
☐	☒	Antibodies
☒	☐	Eukaryotic cell lines
☒	☐	Palaeontology and archaeology
☒	☐	Animals and other organisms
☐	☒	Human research participants
☐	☒	Clinical data
☒	☐	Dual use research of concern

Methods

n/a	Involved in the study	
☒	☐	ChIP-seq
☒	☐	Flow cytometry
☒	☐	MRI-based neuroimaging

Antibodies

Antibodies used	IHC staining for CD3 using the clone LN10 was performed on the Leica Bond-3 auto staining system (Leica, Deerfield, IL, ready-to-use [1 ug/mL]), using heat-based antigen retrieval, a high pH buffer solution (AR9640; Leica, Bond Epitope Retrieval Solution 2), and a polymer detection system (DS9800; Leica, Bond Polymer Refine Detection). CD3 was reported as the area occupied by CD3+ cells over the total intratumoral stromal area. IHC for CD4 and CD8 was performed using the clones SP35 and SP57, respectively, both manufactured by Ventana (Tucson, AZ, ready-to-use). The evaluation was carried out according to the percentage of cytoplasmic positivity expression. Absolute counting of positively stained cells was performed as evaluation criteria to assess IHC for CD4 and CD8 markers. Staining was blinded assessed by an experienced breast cancer pathologist (FT). PD-L1 expression was assessed by IHC using Ventana SP142 assay (dilution 1:50; Spring Biosience, USA). Immune cells infiltrating tumors with positive staining rate ≥1% were classified as PD-L1-positive. IHC for Ki67 (ready-to-use, Ventana, anti-Ki-67 (30-9)) was performed automatically using an IHC autostainer (BenchMark® XT, Ventana Medical Systems, Inc.). The Ki67 score was calculated obtaining the percentage of positive tumor cells among the total number of tumor cells in each tissue section.
Validation	*Describe the validation of each primary antibody for the species and application, noting any validation statements on the manufacturer's website, relevant citations, antibody profiles in online databases, or data provided in the manuscript.*

Human research participants

Policy information about studies involving human research participants

Population characteristics	We collected specimens from a stage III TNBC patient (estrogen, progesterone and HER2 receptors negativity, Ki67 60%, grade 3) with a 3.5cm right breast cancer mass and node involvement who underwent multiple systemic therapies due to recurrences and metastatic progression over 2,033 days of clinical follow-up.

Recruitment	Translational project
Ethics oversight	The Ethics Committee of the Dexeus Institute of Oncology, Quironsalud Group, Barcelona, approved the study.

Note that full information on the approval of the study protocol must also be provided in the manuscript.

Clinical data

Policy information about clinical studies

All manuscripts should comply with the ICMJE guidelines for publication of clinical research and a completed CONSORT checklist must be included with all submissions.

Clinical trial registration	n/a
Study protocol	n/a
Data collection	From December 2012 to June 2018
Outcomes	n/a
